# The impact of oral carbohydrate-rich supplement taken two hours before caesarean delivery on maternal and neonatal perioperative outcomes -- a randomized clinical trial

**DOI:** 10.1186/s12884-021-04155-z

**Published:** 2021-10-07

**Authors:** Yuanying He, Chunhong Liu, Ying Han, Yun Huang, Jianhong Zhou, Qigui Xie

**Affiliations:** 1 Department of Obstetrics and Gynaecology, The Chinese University of Hong Kong, Hong Kong, SAR China; 2 Department of Gynecology and Obstetrics, Shanghai Tenth People’s Hospital, Tongji University School of Medicine, No. 301, Yanchangzhong Road, Shanghai, 200072 China

**Keywords:** Enhanced recovery after surgery (ERAS), Oral carbohydrate-rich supplement, Caesarean delivery (CD), Homeostatic model assessment of insulin resistance (HOMA-IR), Neonatal glucose level

## Abstract

**Background:**

To evaluate the impact of oral carbohydrate-rich (Ch-R) supplement taken 2 hours before an elective caesarean delivery (CD) on maternal and neonatal perioperative outcomes.

**Methods:**

Ninety pregnant women undergoing elective CD were randomized into the Ch-R group, placebo group and fasting group equally. Participants’ blood was drawn at three time points, before intervention, immediately after and 1 day after the surgery to measure maternal and neonatal biochemical indices. Meanwhile women’s perioperative symptoms and signs were recorded.

**Results:**

Eighty-eight pregnant women were finally included in the study. Women who had drunk Ch-R supplement had lower postoperative insulin level (β = − 3.50, 95% CI − 5.45 to − 1.56), as well as postoperative HOMA-IR index (β = − 0.74, 95% CI − 1.15 to − 0.34), compared with women who had fasted. Additionally, neonates of mothers who were allocated in the Ch-R group also had a higher glucose level, compared with neonates of mothers in the fasting group (β = 0.40, CI 0.17 to 0.62).

**Conclusion:**

Oral Ch-R solution administered 2 hours before an elective CD may not only alleviate maternal postoperative insulin resistance, but also comfort women’s preoperative thirst and hunger, compared to fasting. Additionally, it may increase neonatal glucose level as well.

**Trial registration:**

Chinese Clinical Trial Registry, ChiCTR2000033163.

Data of Registration: 2020-5-22.

**Supplementary Information:**

The online version contains supplementary material available at 10.1186/s12884-021-04155-z.

## Background

The traditional fashion that patients preoperatively fast from midnight to minimum 6 h for solid food and 4 h for fluids before an elective surgery to prevent vomiting and aspiration pneumonia has been challenged by various studies [1, 2]. Therefore, several updated guidelines encourage patients to take clear fluids up to 2 h before an elective operation with strong grade recommendations [3–6].

Accumulated evidences have showed that shortening of the fasting interval will not decrease the pH, or increase the volume of gastric contents related to perioperative complications [7, 8]. Moreover, it could alleviate both preoperative [9] and postoperative [10, 11] discomfort (e.g. nausea and vomiting). Prolonged interval of fasting (e.g. overnight fasting) is detrimental to patients’ postoperative recovery [12]. This was mainly on account of the stress response-related postoperative insulin resistance. Lower insulin resistance was found when oral carbohydrate beverage was taken before operation, compared with fasting or placebo drinks in several trials [13–15].

The European Society of Anaesthesiology recommended clear fluids, including pulp-free juice, tea, and coffee to drink up to 2 hours before an elective caesarean delivery (CD) [5]. Moreover, guidelines for antenatal and preoperative care in caesarean delivery published by Enhanced Recovery After Surgery (ERAS) Society in 2018 made the same recommendation with strong grade. Nevertheless, the grade of the recommendation for oral carbohydrate fluid supplementation taken 2 hours before CD was weak due to the low-level evidence [16]. Our study is aiming to address the impact of preoperative carbohydrate fluid supplementation on postoperative insulin resistance and discomfort in pregnant women undergoing elective CD, as well as on neonatal glucose level.

## Methods

### Study design

This was a randomized controlled trial involving oral administration of carbohydrate supplement before elective CD, compared with preoperative fasting and preoperative water intake, respectively, in order to determine the impact of preoperative carbohydrate supplement on pregnant women’s postoperative blood glucose level, insulin level, insulin resistance, perioperative discomfort, as well as neonates’ blood glucose level.

### Participants

Women with a singleton pregnancy, who were planned to receive an elective CD at Shanghai Tenth People’s Hospital were recruited. The exclusion criteria were women with gestational diabetes mellitus according to the International Association of Diabetes and Pregnancy Study Groups (IADPSG) [17], or gestational hypertension according to the American College of Obstetricians and Gynecologists (ACOG) diagnostic criteria [18]. Women with overweight or obese pre-pregnancy BMI according to the Chinese standards (i.e. BMI ≥ 24), or having hepatic disease (including intrahepatic cholestasis during pregnancy) were also excluded. The purposes and details of the study were notified to the pregnant women. Meanwhile, written consents were obtained from them before recruitment. The trial was approved by the local ethics committee at Shanghai Tenth People’s Hospital and carried out in accordance with the Helsinki Declaration.

### Intervention

All the participants were randomized with an allocation ratio of 1:1:1 by an independent research assistant who was not involved in the following process, via a random number generator tool in Microsoft Excel (Microsoft, Redmond, WA). Group allocations were placed in sequentially numbered opaque sealed envelopes. After obtaining consent forms, pregnant women were included in the trial. Both the surgeons and the researchers were blinded to the treatment assignment.

The first surgery started at 8:30 AM and subsequent surgeries followed. All the surgeries initialled before 12:00 PM. Pregnant women allocated to the Ch-R group received 400 ml oral carbohydrate-rich (Ch-R) solution (Shu Yu™, Chongqing, China, supplementary note 1) 2 hours before the CD. The Ch-R solution had the same appearance but different taste compared with clear water, and contained 12.5 g carbohydrate and 213 kJ caloric per 100 ml. Pregnant women allocated to the placebo group took a placebo (400 ml clear water) 2 hours before the CD. Pregnant women allocated to the fasting group abstained from solid food or fluids before the CD. The last solid meal was given to all women in the evening before the surgery. Oral fluids were stopped by 22:00 on the day before the surgery day. None of the women received pre- or intraoperative intravenous infusion of glucose solution. During the CD, 1000–1500 ml of crystalloids was intravenously infused. After surgery, 1500–2000 ml of crystalloids, including 500 ml 5% glucose solution was infused when returning to the ward. Women were allowed to take liquid diet 6 hours after the operation. The manner of the CD was transverse incision of the lower uterine segment under spinal anaesthesia.

### Exposure variables

Maternal BMI at delivery was calculated as weight (kg) / height^2^ (m^2^), where weight and height were measured when patients were admitted for delivery. Gestational age (GA) was recorded on the day of operation. The duration of surgery was timed from disinfection until patients left the surgery room. Meanwhile, neonatal birth weight and sex were recorded.

### Outcome variables

Pregnant women’s plasma glucose and insulin levels were examined to assess the impacts on glucometabolic. Blood samples were delivered to the laboratory immediately. The assays were conducted within 2 hours by using hexokinase and electrochemiluminescence methods, respectively. The primary outcome was pregnant women’s postoperative insulin resistance evaluated by the homeostatic model assessment of insulin resistance (HOMA-IR) index, which was calculated according to the formula: HOMA-IR = blood glucose level (mmol/L) x blood insulin level (mIU/L) / 22.5. Blood samples before intervention were taken from every woman at 2 hours before the CD (before preoperative carbohydrate-rich solution or water was taken), while blood samples immediately after and 1-day after the CD were obtained when they returned to the ward from the surgery room immediately, and 6:00 AM on the day after the surgery day, respectively.

Pregnant women’s perioperative symptoms and signs were also accessed as secondary outcomes. The questions were asked and recorded by an independent research assistant. Women were asked to rate their thirst and hunger on a 10-cm visual analogue scale, with 0 representing “not thirsty/hungry at all” and 10 representing “extremely thirsty/hungry” on arrival at the surgery room. Questions about the occurrences of intra- or postoperative nausea and vomiting were asked 24 h after the CD. Time of flatus started when women arrived at the ward after the CD. Oral temperatures were measured four times 1 day in the first 48 h after the CD, and postoperative fever was diagnosed once temperature was equal to or higher than 37.5 °C. Women were discharged on the fourth day after the CD, unless complication existed.

For neonatal outcomes, 1-min Apgar score and blood glucose level were recorded. Capillary blood glucose level was measured on heel within 20 min after delivery.

### Sample size

The sample size was calculated based on the HOMA-IR outcomes of a pilot study. Ten pregnant women in total were stratified into Ch-R group and fasting group equally. As a result, 17 patients in the fasting group would achieve a 91% power to detect a difference of − 0.6 to the CHR group with a significance level (alpha) of 0.05 using a two-sided t-test. Accounting for 20% loss to complete the protocol, at least 23 patients per group were required in the study.

### Statistical methods

Continuous variables were expressed as median (Q1-Q3) since the data of each group were not normally distributed with a small sample size. The Kruskal-Wallis test was carried out, followed by post-hoc pairwise comparison with Bonferroni correction if the null hypothesis was rejected. Dichotomous variables were expressed as number (percentage), and analysed via the χ^2^ test or Fisher’s Exact test when appropriate.

Furthermore, several traits were adjusted for covariates using linear regression as follows: maternal postoperative biochemical indices were adjusted for relevant preoperative variables, age, BMI and duration of the operation, while neonatal blood glucose level was adjusted for sex and birth weight. Two dummy variables were used to code for the categories, “Ch-R group” and “placebo group”. Each group was compared with the reference category “fasting group”. Meanwhile, repeated measures ANOVA were performed to test the differences over time, as well as the interaction between groups and time.

SPSS version 22 software (SPSS, Chicago, IL) was used to perform all statistical analysis. GraphPad Prism 5.0 software (GraphPad Software, Inc., La Jolla, CA, USA) was employed to plot the graphs. *P* < 0.05 was considered to indicate a statistically significant difference.

## Results

### Participant characteristics

**Fig. 1:**
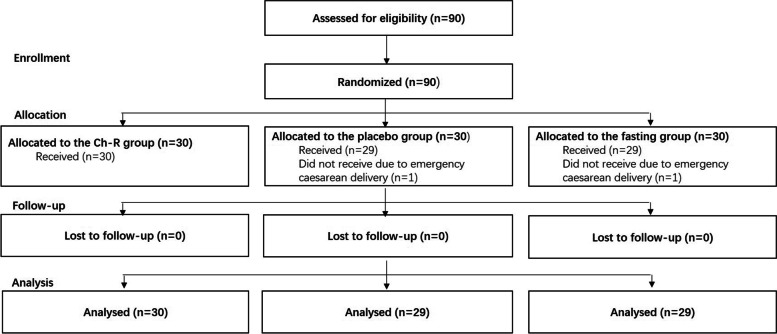
CONSORT diagram for the participants

The study was carried out between 2020 and 05-27 and 2020-07-26. In total, 90 pregnant women were randomized into three groups equally. Two patients were excluded due to emergency CD on the night before the expected surgery day (Fig. 1). Among the 88 pregnant women, the median maternal BMI at birth was 27.05 kg/m^2^, and the median duration of operation was 49 min. The median birth weight was 3380 g. There was no significant difference found in the 88 mother-neonate dyads’ demographics and anthropometrics between groups (Table 1).

**Table 1:** Maternal and neonatal characteristics

	Ch-R group (*n* = 30)	Placebo group (*n* = 29)	Fasting group (*n* = 29)	P
*Maternal characteristics*				
Age (y)	30.0 (28.8–34.0)	31.0 (29.5–34.5)	32.0 (29.0–35.5)	0.756
BMI (kg/m^2^)	26.9 (25.2–28.0)	27.1 (24.3–28.6)	27.7 (25.6–30.1)	0.332
GA (wks)	39.0 (38.8–39.3)	39.1 (38.9–39.7)	39.0 (38.6–39.3)	0.257
Duration of surgery (min)	49.0 (41.8–55.3)	49.00 (45.0–60.0)	49.5 (42.0–55.5)	0.436
*Neonatal characteristics*				
Sex male	14 (46.7)	12 (41.4)	16 (55.2)	0.569
Birthweight (g)	3405 (3227–3750)	3380 (3105–3635)	3340 (3130–3600)	0.581

Data are expressed as median (Q1-Q3) or n (%). Women in the Ch-R group received 400 ml oral Ch-R solution 2 hours before the surgery. Women in the placebo group took 400 ml clear water 2 hours before the surgery. Women in the fasting group received preoperative fasting. *Ch-R* carbohydrate-rich, *BMI* body mass index, *GA* gestational age

### Biochemical indices

Pregnant women who had drunk Ch-R solution had lower immediate postoperative insulin level, as well as HOMA-IR index, compared with their counterparts in the placebo group and fasting group, respectively. However, the maternal immediate postoperative glucose level exhibited no difference between groups. On the other hand, neonates delivered from mothers who had drunk the Ch-R solution had a higher glucose level than those from mothers who had fasted before CD (Table 2).

**Table 2:** Maternal and neonatal biochemical indices

	Ch-R group (*n* = 30)	Placebo group (*n* = 29)	Fasting group (*n* = 29)	P
*Maternal biochemical indices*				
Before intervention				
Glucose (mmol/L)	4.2 (4.0–4.4)	4.1 (4.0–4.6)	4.2 (4.0–4.3)	0.800
Insulin (mU/L)	11.3 (8.9–14.8)	11.5 (9.0–14.6)	11.6 (9.0–14.6)	0.976
HOMA-IR	2.1 (1.6–2.9)	2.2 (1.6–2.9)	2.2 (1.6–2.9)	0.988
Postoperative				
Glucose (mmol/L)	4.2 (3.6–4.7)	4.3 (3.9–4.7)	4.4 (3.9–4.8)	0.395
Insulin (mU/L)	4.6 (2.8–7.6) ^*#^	8.0 (4.5–13.3) ^*^	8.6 (5.0–11.5) ^#^	0.004
HOMA-IR	0.9 (0.5–1.5) ^*#^	1.6 (0.8–2.6) ^*^	1.6 (0.9–2.3) ^#^	0.004
1-day after CD				
Glucose (mmol/L)	3.9 (3.7–4.2)	3.9 (3.5–4.1)	3.7 (3.1–4.2)	0.313
Insulin (mU/L)	2.9 (1.8–4.9)	1.8 (1.3–2.8)	2.7 (1.8–4.3)	0.050
HOMA-IR	0.5 (0.3–0.8)	0.3 (0.2–0.5)	0.5 (0.3–0.7)	0.059
*Neonatal biochemical indices*				
Glucose (mmol/L)	3.2 (2.9–3.7) ^*^	3.0 (2.8–3.3)	3.0 (2.8–3.2) ^*^	0.032

Data are expressed as median (Q1-Q3). Women in the Ch-R group received 400 ml oral carbohydrate-rich solution 2 hours before the surgery. Women in the placebo group took 400 ml clear water 2 hours before the surgery. Women in the fasting group received preoperative fasting. Biochemical indices before intervention were measured at 2 hours before the surgery (before preoperative carbohydrate-rich solution or water was taken), while indices immediately after and 1-day after CD were obtained when they returned to the ward from the surgery room immediately and 6:00 AM on the day after the surgery day, respectively. *HOMA-IR* homeostatic model assessment of insulin resistance, *CD* caesarean delivery. *# *P* < 0.05 for between group comparison

Furthermore, with the adjustments for maternal age, BMI, the duration of surgery and the insulin level before the intervention, women in the Ch-R group had a lower postoperative insulin level than the fasting group (β = − 3.50, confidence interval [CI] -5.45 to − 1.56). Similarly, they also had a lower postoperative HOMA-IR index (β = − 0.74, CI − 1.15 to − 0.34). Additionally, with adjustments for neonatal sex and birth weight, neonates from mothers allocated in the Ch-R group still had a higher glucose level (β = 0.40, CI 0.17 to 0.62, Table 3).

**Table 3:** Impacts of preoperative Ch-R supplements on maternal postoperative biochemical indices and neonatal glucose level

	Ch-R group				Placebo group				Fasting group
	Unadjusted		Adjusted		Unadjusted		Adjusted		
	β (95% CI)	P	β (95% CI)	P	β (95% CI)	P	β (95% CI)	P	
*Maternal biochemical indices*									
Glucose (mmol/L)	−0.18 (− 0.52 to 0.16)	0.292	− 0.15 (− 0.49 to 0.18)	0.363	− 0.06 (− 0.40 to 0.29)	0.750	− 0.11 (− 0.44 to 0.23)	0.527	Reference
Insulin (mU/L)	−3.4 (−5.7 to −1.2)	0.003	−3.5 (− 5.5 to − 1.6)	0.001	−0.07 (−2.4 to 2.2)	0.951	0.08 (− 1.8 to 2.0)	0.931	Reference
HOMA-IR	−0.72 (− 1.2 to − 0.24)	0.004	−0.74 (− 1.2 to − 0.34)	< 0.001	−0.01 (− 0.49 to 0.48)	0.975	−0.02 (− 0.42 to 0.38)	0.913	Reference
*Neonatal biochemical indices*									
Glucose (mmol/L)	0.37 (0.15 to 0.59)	0.001	0.40 (0.17 to 0.62)	0.001	0.09 (−0.13 to 0.32)	0.413	0.12 (−0.10 to 0.35)	0.288	Reference

Data are expressed as β [95% confidence interval (CI)]. The Ch-R group and the placebo group were respectively compared with the fasting group. Women in the Ch-R group received 400 ml oral Ch-R solution 2 hours before the surgery. Women in the placebo group took 400 ml clear water 2 hours before the surgery. Women in the fasting group received preoperative fasting. Maternal postoperative biochemical indices were adjusted for relevant preoperative indices, age, BMI and the duration of the surgery. Neonatal glucose level was adjusted for sex and birth weight. *Ch-R* carbohydrate-rich, *CRP* C-reactive protein, *HOMA-IR* homeostatic model assessment of insulin resistance

**Fig. 2:**
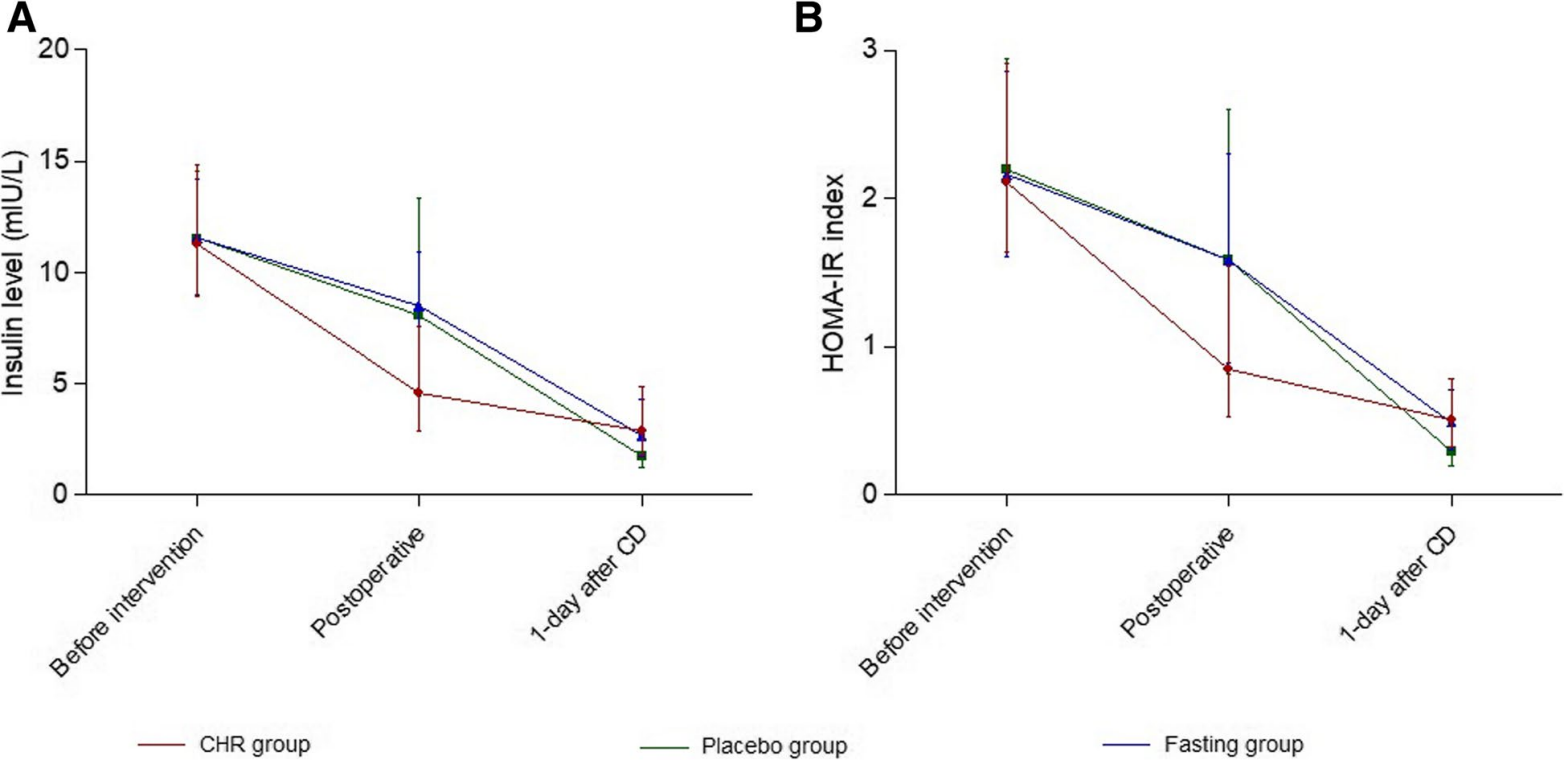
Maternal insulin level and HOMA-IR index at different time points in different groups. Data were expressed as median with whiskers as inter-quartile range. Women in the Ch-R group received 400 ml oral carbohydrate-rich solution 2 hours before the surgery. Women in the placebo group took 400 ml clear water 2 hours before the surgery. Women in the fasting group received preoperative fasting. Biochemical indices before intervention were measured at 2 hours before the surgery (before preoperative carbohydrate-rich solution or water was taken), while indices immediately after and 1-day after the surgery were obtained when they returned to the ward from the surgery room immediately and 6:00 AM on the day after the surgery day, respectively. **A** Maternal insulin levels at different time points in different groups; **B** Maternal HOMA-IR index at different time points in different groups. CD, caesarean delivery; HOMA-IR, homeostatic model assessment of insulin resistance

On the other hand, both the unadjusted maternal insulin level and HOMA-IR index descended over time (Table 4, Fig. 2). The interaction between time and group exhibited significant, regardless of maternal age, BMI and the duration of surgery (Table 5).

**Table 4:** Repeated measures ANOVA on maternal biochemical indices

	Unadjusted				Adjusted			
	SS	df	MS	P	SS	df	MS	P
Insulin								
Group	25.7	2	12.9	0.663	8.5	2	4.3	0.846
Time	3439.7	2	1719.8	< 0.001	25.3	2	12.6	0.249
Group × Time	231.0	4	57.7	< 0.001	226.1	4	56.5	< 0.001
HOMA-IR								
Group	1.4	2	0.72	0.578	0.82	2	0.41	0.691
Time	128.8	2	64.4	< 0.001	1.0	2	0.52	0.254
Group × Time	9.5	4	2.4	< 0.001	9.2	4	2.4	< 0.001

Indices were adjusted for maternal age, BMI and the duration of the surgery. *SS* type III sum of squares, *df* degree of freedom, *MS* mean square, *HOMA-IR* homeostatic model assessment of insulin resistance

### Perioperative symptoms and signs

In the preoperative assessment, women who had drank Ch-R solution were found less thirsty and hungry. Meanwhile, women who had received preoperative water felt less thirsty compared with those who had fasted (Table 5).

**Table 5:** Maternal and neonatal perioperative symptoms and signs

	Ch-R group (*n* = 30)	Placebo group (*n* = 29)	Fasting group (*n* = 29)	P
*Maternal outcomes*				
Preoperative thirst	0 (0–0) ^*^	0 (0–0) ^#^	3 (0–3) ^*#^	< 0.001
Preoperative hunger	0 (0–0) ^*#^	0 (0–3) ^*^	1 (0–3) ^#^	0.002
Perioperative nausea	1 (3.3)	3 (10.3)	1 (3.5)	0.525
Perioperative vomiting	1 (3.3)	2 (6.9)	0 (0.0)	0.540
Aspiration pneumonia	0 (0)	0 (0)	0 (0)	–
Time of flatus (h)	26.5 (22.8–30.25) ^*^	35.0 (27.5–42.0) ^*#^	29.0 (25.0–32.5) ^#^	0.001
Length of stay (d)	4 (4–4)	4 (4–4)	4 (4–4)	0.158
Postoperative fever	9 (30.0)	10 (34.5)	11 (37.9)	0.812
*Neonatal outcome*				
Apgar score	10 (10–10)	10 (10–10)	10 (10–10)	0.362

Data are expressed as median (Q1-Q3) or n (%). Women in the Ch-R group received 400 ml oral carbohydrate-rich solution 2 hours before the surgery. Women in the placebo group took 400 ml clear water 2 hours before the surgery. Women in the fasting group received preoperative fasting. Thirst and hunger were accessed on a 10-cm visual analogue scale, with 0 representing “Not thirsty/hungry at all” and 10 representing “Extremely thirsty/hungry”. Time of flatus started when patients arrived at the ward after the surgery. *# *P* < 0.05 for between group comparison

There was no significant difference in terms of adverse anaesthetic outcomes (i.e. perioperative nausea, vomiting and aspiration pneumonia) found between the groups. Women who had drunk water farted later than their counterparts in the other two groups. The length of stay after the surgery, the incidence of postoperative fever, as well as neonatal 1-min Apgar score showed no difference between the groups (Table 5).

## Discussion

With evidence accumulated, fasting guidelines in various countries were updated to permit clear liquid up to 2–3 h before surgery. Randomized controlled trials and meta-analysis have demonstrated that preoperative fluid intake would improve the feeling of well-being and alleviate thirst and dryness of the mouth [10, 12]. A specially designed high-carbohydrate contained beverage administrated in the preoperative period is recommended due to the reduction of the catabolic stress response to the surgery [14]. The ERAS society encouraged oral carbohydrate fluid supplementation 2 hours before caesarean delivery to nondiabetic women in the guidelines for antenatal and preoperative care in caesarean delivery in 2018. Nevertheless, the grade of the recommendation was weak due to the low-level evidence [16]. Our finding showed that the administration of oral carbohydrate-rich solution 2 hours before caesarean delivery may not only reduce the maternal postoperative insulin resistance, but also increase the neonatal glucose level.

Crenshaw et al. found that both actual and instructed fasting durations prior to the CD were longer than national guidelines in US. The average thirst and hunger scores were 5 and 4, respectively [19]. Another previous study showed that the duration of fluid fasting in women undergoing elective CD was correlated with thirst scores and urine osmolality [20]. Meanwhile, latest findings from Wendling et al. found that a higher-dose carbohydrate beverage consumed preoperatively resulted in superior well-being compared to fasting, which was consistent with our findings [21]. However, considering patients were not blind in our study, bias may have occurred accordingly.

A recently published study found that carbohydrate drinks ahead of CD reduced the incidence of urinary ketones immediately prior to surgery [22]. Our study used HOMA-IR index to access the metabolic state. The impact of preoperative carbohydrate supplement on the reduction of postoperative insulin resistance has been found in other types of surgeries [2, 15]. Insulin resistance is one of the most fundamental reactions to injury and stress. Previous studies in elective surgeries suggested that insulin resistance was associated with prolonging recovery and postoperative complications, in particular infections [23]. The main objective of the preoperative carbohydrate supplement is to mimic a change in metabolism that generally occurs due to breakfast intake. It will elicit an endogenous insulin release that turns off the overnight fasting state of the metabolism in order to improve postoperative recovery [24].

A cross-sectional study involved approximate 3000 Chinese adults showed the 75th (1.4) and 90th (2.0) percentiles of HOMA-IR index were appropriate to be the optimal cut-off points to discriminate dysglycemia and Type II diabetes, respectively [25]. The medians of HOMA-IR index before intervention in our study exceeded the 90th percentiles in all three groups, which implied a high risk of insulin resistance, and is detrimental to postoperative recovery. Nevertheless, only the median of HOMA-IR index in Ch-R group restored to the level less than 75th percentile immediately after the CD, which may reduce the risk. All participants’ HOMA-IR indices descended to normal level on the day after the surgery day. This may be partly influenced by the postoperative infusion of 500 ml 5% glucose solution after the surgery.

Li et al. found that shorter fasting interval (solid food < 8 h and clear fluid < 2 h) reduced the incidence of hypoglycaemia and acidosis in neonates. However, the incidence of vomiting of women increased while the fasting interval of solid food reduced to less than 6 hours [26]. On the other hand, intravenous glucose solution administered during the CD has increased both the maternal and neonatal glucose level [27]. We noticed that the mean umbilical cord blood glucose level of those who had drunk preoperative oral carbohydrate beverage in Wendling et al.’s study was relative higher (*p* > 0.05) [21]. Fortunately, with a larger sample size, a significant difference was obtained in our study. Our finding suggested that the oral carbohydrate-rich solution administered 2 hours prior to CD may independently increase the neonatal glucose level, apart from increasing the risks of maternal hyperglycaemia, vomiting or nausea.

There were several limitations in our study. The fasting-CD intervals, as well as the contents of last meals varied among individuals, and were not recorded and standardized in the study. On the other hand, with such a small sample size, we are incapable to address the impact of preoperative oral carbohydrate-rich solution on adverse anaesthetic outcomes.

## Conclusions

Oral Ch-R solution administered 2 hours before an elective CD may not only alleviate maternal postoperative insulin resistance, but also comfort women’s preoperative thirst and hunger, compared to fasting. Additionally, it may increase neonatal glucose level as well. Our findings support the recommendation on carbohydrate-rich solution prior to elective CD.

## Supplementary Information

**Additional file 1.**

## Data Availability

The datasets used and analysed during the current study available from the corresponding author on reasonable request. Proposals should be directed to: cykstar@163.com. To gain access, data requestors will need to sign a data access agreement.

## Abbreviations

CD: Caesarean delivery;
ERAS: Enhanced recovery after surgery
Ch-R: Carbohydrate-rich
GA: Gestational age
HOMA-IR: Homeostatic model assessment of insulin resistance
