# Supplementary material for: The impact of oral carbohydrate-rich supplement taken two hours before caesarean delivery on maternal and neonatal perioperative outcomes -- a randomized clinical trial
Source: BMC Pregnancy Childbirth. 2021 Oct 7;21:682. doi: 10.1186/s12884-021-04155-z (PMC8495981; doi:10.1186/s12884-021-04155-z)
Supplement: Supplementary file 1 — Additional file 1. [file 12884_2021_4155_MOESM1_ESM.docx]

Supplementary note 1 The ingredients of oral carbohydrate-rich solution is listed below:

Water, maltodextrin, crystalline fructose, potassium citrate, citric acid, sodium citrate, sodium benzoate, stevioside, flavor.
